# When Aesthetics, Surgery, and Psychology Meet: Aesthetic Nasal Proportions in Patients Having Rhinoplasty and Normal Adults

**DOI:** 10.1055/s-0036-1579658

**Published:** 2016-02-24

**Authors:** Mohsen Naraghi, Mohammad Atari, Hossein Asadollahi

**Affiliations:** 1 Division of Rhinology and Facial Plastic Surgery, Department of Otorhinolaryngology–Head and Neck Surgery, Tehran University of Medical Sciences, Tehran, Iran; 2 Otorhinolaryngology Research Center, Tehran University of Medical Sciences, Tehran, Iran; 3 Rhinology Research Society, Tehran, Iran; 4 Department of Psychology, University of Tehran, Tehran, Iran; 5 Medical Sciences Branch, Islamic Azad University, Tehran, Iran

**Keywords:** facial plastic surgery, facial aesthetics, rhinoplasty, psychology

## Abstract

The aesthetic nasal proportions have played a significant role in rhinoplasty practice. On the other hand, psychological variables also play a crucial role in rhinoplasty. It is of paramount importance for facial plastic surgeons to consider both sides to achieve a more satisfactory outcome. The present study aimed to compare aesthetic nasal proportions between primary rhinoplasty candidates and a demographically matched control group to determine whether patients having rhinoplasty have different aesthetic nasal proportions compared with healthy adults who are not interested in rhinoplasty. Sixty patients having rhinoplasty were selected consecutively from a surgical clinic. A control group ( *n* = 60) with the same demographic characteristics was selected. Photographs were taken using a digital camera on a fixed zoom setting. All images were captured at a distance of 1.5 m. Frontal and right lateral views were used to compare nasolabial angle, nasofrontal angle, nasofacial angle, alar width, intercanthal distance, nasal length, and width-to-length ratio. Independent *t* tests were used for comparisons. Independent *t* tests verified that nasofrontal angle, nasal length, and width-to-length ratio were significantly different between the two groups ( *p* < 0.01). Effect sizes ranged between 0.11 and 0.69. Aesthetic proportions were not significantly different in four factors. Nasolabial angle, nasofacial angle, alar width, and intercanthal distance were not different ( *p* > 0.05). Four major aesthetic nasal proportions were statistically similar in a group of patients having rhinoplasty and a control group with no interest in rhinoplasty. Surprisingly, the patients having rhinoplasty showed a mean width-to-length ratio closer to aesthetic ideal. Therefore, applying for rhinoplasty may have strong psychological reasons (e.g., body dysmorphic symptoms) compared with realistic aesthetic appraisals.

The obsession with facial beauty is not limited to modern culture. Several studies have suggested that facial attractiveness is comparatively independent of culture. 1 2 Attractive faces activate the reward centers in the brain, 3 they motivate sexual behavior and the development of same-sex alliances, 4 5 and they elicit positive behaviors in various situations. 6 As a result, it is not surprising that philosophers, scientists, and even lay people have long puzzled over what makes a face more attractive and why we hold the preferences we have. 7 It might also clarify the reason behind the popularity of facial plastic surgeries.

Historically, the face has been considered the personification of one's soul. From the social point of view, it is the representation of the person's identity. In fact, in many countries it is mandatory after a fixed number of years to change the picture on the identity document to verify how the face has changed. 8 This practice represents the importance of the face in social interactions and law.

All facial parts are of absolute importance for the perception of facial beauty. However, the nose has a special importance because it occupies the central position in the face. The nose is also the most prominent anatomic part of the face; it cannot be hidden easily. Thus, the nose's aspect is critical not only for the anatomy of the face but also because this organ is one of the factors that can disturb one's body image. 9

People's awareness of the fact that the nose is a crucial element in facial beauty and the emphasis on beauty in mass media make aesthetic rhinoplasty one of the most frequently requested aesthetic operations. It has been suggested that cosmetic surgery is essentially body image surgery, 10 and facial plastic surgery will particularly enhance body image, quality of life, 11 personality perceptions, 12 and perceived age. 13 On the other hand, a review of the evidence concluded that it was scientifically premature to assume that cosmetic surgery necessarily leads to direct psychological benefits. 14 As a result, a general lack of well-designed research seems to be present, particularly in the range of possible psychological outcomes following cosmetic surgeries. 15

The psychological aspects of aesthetic rhinoplasty have started to attract clinicians' and researchers' attention in the past few decades. Generally, it has been shown that patients having aesthetic rhinoplasty show stronger symptoms of psychopathology in comparison with patients having functional surgery. 16 17 18 19 The average self-esteem score of these patients has been reported to be lower than control populations, 20 and a higher interest in aesthetic rhinoplasty has been correlated with lowered body appreciation. 21 Body dysmorphic disorder (BDD) has also attracted much attention from the researchers. A high prevalence of BDD has been reported among patients who undergo facial plastic surgeries, particularly rhinoplasty. 22 23 Patients who suffer from BDD are extremely dissatisfied with their physical appearance. BDD is defined as preoccupation with an imagined defect in one's appearance. 24

Patients with BDD are not objective about their appearance. They are not satisfied with their appearance, although in many cases their appearance is normal by outside judgment. A very conceptual characteristic in patients with BDD is their lack of objectivity. Patient with BDD whose obsession is toward the nose simply cannot be objective about their nasal shape. They are preoccupied with their nose and spend much time ruminating over a defect that is unrealistic and in many cases nonexistent. But what makes a nose objectively more attractive? Are there any pre-established aesthetic standards?

Researchers and clinicians have presented some standards for facial aesthetics; however, these proportions are relative and might vary slightly across different cultures. Nasal proportions that are closer to these “ideals” cause the nose to be perceived as more beautiful.

The aim of rhinoplasty is to create a nose that is aesthetically pleasing to the patient without compromising nasal function. 25 In achieving this aim, it is essential for rhinoplasty surgeons to have a thorough knowledge of nasal aesthetics. The facial aesthetic standards have been used in rhinoplastic surgery practice. These standards are based on reference to an artistic ideal of beauty without any supporting population-based studies. 26 27 If the aim of an individual rhinoplasty is the creation of a beautiful nose, then the use of the ideal aesthetic as a standard is appropriate. 28 If, however, the patient's aim is to have a normal-looking nose, then the “ideal aesthetic” may seem an inappropriate standard. Given that rhinoplasty is very often performed to correct shape changed by trauma, it may be false to assume that all patients necessarily prefer beauty to correction of these specific shape-related changes. 29

Facial aesthetic ideals are both culturally variable and time sensitive. The “ski-slope nose” with marked cephalic tip rotation that was desirable in the 1950s is less desired now. The lack of representative population norms in facial aesthetics means that the artistic ideals constitute the most available reference. 30 31 32 33 34 35 Although small numbers of population cohort studies have been performed in the study of the ethnic nose, little is known of the aesthetic standards of nasal proportions in the general population. 36 37

The present study aimed to identify the role of facial aesthetic proportions in the interest in aesthetic rhinoplasty by comparing the proportions between primary rhinoplasty candidates and a demographically matched control group. It was hypothesized that if patients evaluate their nasal shape realistically, then their aesthetic facial proportions should be aesthetically less pleasing than the control group. The less realistic evaluations may be representative of body dysmorphic traits.

## Methods

### Participants

Sixty patients having rhinoplasty were consecutively selected from a private surgical clinic. The control group consisted of 60 people who were matched with the patients on the basis of age, sex, general health, and history of nasal trauma. The baseline characteristics of cases and controls are presented in Table 1 .

**Table 1:** Demographic characteristics of cases and controls

	Patients having rhinoplasty	Control group
*n*	60	60
Mean age, y (SD)	26.53 (7.42)	26.57 (6.20)
Sex		
Male	12	12
Female	48	48
Nasal trauma		
Yes	0	0
No	60	60

Abbreviation: SD, standard deviation.

### Measures

#### Nasolabial Angle

The nasolabial angle is defined as the angle between the line drawn through the midpoint of the nostril aperture and a line drawn perpendicular to the Frankfurt horizontal and intersecting the subnasale. An arbitrary range of 90 to 115 degrees for the nasolabial angle is usually stated in the literature. 38

#### Nasofrontal Angle

The nasofrontal angle is located between a line drown from the radix tangential to the glabella and a second line from the same point tangential to the nasal tip. Angles in an aesthetically pleasing profile average from 115 to 130 degrees. This aesthetic feature plays a very important role in facial beauty. 39

#### Nasofacial Angle

The nasofacial angle is formed by drawing a vertical line tangent to forehead at the glabella and tangent to the chin at the pogonion so that a line drawn along the nasal dorsum intersects it. 40 The ideal nasofacial angle is between 30 and 40 degrees. Powell and Humphreys also suggested that the female profile be at the lower end of the ideal range and the male profile at the upper end. 35

#### Alar Width

The alar width is defined as the distance between the outermost points of alar bases at the junction of the alar base and the lip. 41 A large study on rhinoplasty candidates reported that alar width varied between 25 and 38 mm preoperatively.

#### Intercanthal Distance

The intercanthal distance is the distance between the medial canthi of the eyes. It usually ranges from 30 to 36 mm. 42 The neoclassical facial canon states that the alar width and the intercanthal distance should be approximately equal. 43

#### Nasal Length

The nasal length is defined as the maximum distance from radix to nasal tip (any part above the columella). The mean value of nasal length has been reported to be around 57.5 mm before rhinoplasty. 41

#### Width-to-Length Ratio

The width-to-length ratio is calculated as the alar width divided by the length of the nose (nasion–pronasion). The width-to-length ratio of the Caucasian nose is widely quoted by many authors to be 0.7. 43

### Procedure

The nature of the study was explained for all participants, and once they provided consent, photographs were obtained. The photographs were taken using a digital camera on a fixed zoom setting. All images were captured at a distance of 1.5 m. Frontal and right lateral views were taken and digitally stored as black-and-white JPEG files. The same procedure was performed for the control group.

### Ethical Considerations

Ethical approval was obtained from the relevant ethics committee. All the participants completed written consent forms before the photos were taken.

### Statistical Analysis

All the tests were two-tailed, and *p* < 0.05 was considered statistically significant. Additionally, Levene's test was performed to assess the equality of variances. Independent *t* tests were used to compare the means of the two groups. Statistical analysis was performed using Statistical Package for the Social Sciences (version 21.0; SPSS, Inc., Chicago, Illinois).

## Results

Most of the cases were women (80%). No significant difference was observed between the case and the control group in demographic characteristics. Age, female-to-male ratio, and history of nasal trauma were matched and had no statistically significant differences ( *p* > 0.05).

Seven aesthetic features of the aesthetic nasal proportions were compared using seven independent *t* tests and seven effect size indices. The results of the comparisons are presented in Table 2 .

**Table 2:** Comparisons of aesthetic facial proportions between patients having rhinoplasty and control group

Aesthetic feature	Patients having rhinoplasty, M (SD)	Control group, M (SD)	*t* Test statistic	*p* Value	Effect size ( *d* )
Nasolabial angle	100.22 (9.32)	101.76 (9.48)	0.89	0.37	0.16
Nasofrontal angle	147.82 (6.12)	142.97 (6.99)	4.04	<0.01	0.69
Nasofacial angle	32.28 (3.20)	32.61 (2.74)	0.62	0.53	0.11
Alar width	36.21 (3.23)	36.72 (2.91)	0.92	0.36	0.17
Intercanthal distance	31.77 (2.62)	31.39 (3.05)	0.73	0.47	0.13
Nasal length	44.05 (2.85)	42.31 (2.16)	3.78	<0.01	0.65
Width-to-length ratio	0.82 (0.08)	0.86 (0.05)	3.28	<0.01	0.57

Abbreviations: M, mean; SD, standard deviation.

## Discussion

Generally, rhinoplasty is a complicated surgery that requires multidisciplinary skills. The surgeon should not perceive rhinoplasty as merely a simple cosmetic procedure. Nasal anatomy, anesthesia, surgical skills, aesthetic perception, psychological evaluation, and sociological considerations have been proved to be involved in a successful rhinoplasty. These factors do not affect the postoperative outcome independently, though. In particular, the subjective factors are correlated in a highly complex manner. In this study, the aesthetic and psychological aspects of rhinoplasty were analyzed. The primary aim of the present study was to identify the role of facial aesthetic proportions in the interest in aesthetic rhinoplasty by comparing the facial aesthetic proportions between rhinoplasty candidates and a demographically matched control group.

It has been decades since facial aesthetic proportions were incorporated into the aesthetic surgical procedures. Many studies have presented aesthetically ideal proportions as a sample for plastic surgeons. However, a quite fundamental question has remained unanswered thus far: what do aesthetic nasal proportions imply? In other words, do ideal aesthetic nasal proportions guarantee self-perceived attractiveness? The empirical evidence from psychology suggests that having high body esteem and positive body image is matter of subjectivity. Moreover, the aesthetic judgment “style” of the patient may play a role in the perception and interpretation of nasal beauty—that is, sometimes aesthetic judgments of the surgeon and the patient on details of nasal beauty are not in agreement. 44 This notion may become clearer by the very definition of BDD. As stated before, BDD biases the patient's attention toward an imaginary defect in appearance. 45 Therefore, the objective measurement of facial aesthetic proportions may not be as important as subjective body esteem. In the case of BDD, aesthetic measurement may be considered irrelevant because the patient does not rely on reality in assessing his or her body.

Seven facial aesthetic proportions were compared between a sample of patients having rhinoplasty and a control group who were not interested in rhinoplasty. Quite surprisingly, the aesthetic proportions were not significantly different in four factors. Nasolabial angle, nasofacial angle, alar width, and intercanthal distance were not different between rhinoplasty candidates and the control group. Nasofrontal angle, nasal length, and width-to-length ratio were different between cases and controls. The control group had an aesthetically better nasofrontal angle; however, the mean width-to-length ratio was closer to ideal in the patients having rhinoplasty. The nasofrontal angle is important in the study of forehead projection, particularly in the glabella area. In profile, the position of the glabella dictates how deep the nasion should be. It has been suggested that the ideal nasofrontal angle should be from 115 to 130 degrees. 35 This range is narrower than the results of both groups in the present study, and the average angle was more obtuse than the suggested ideal. It is therefore essential to ensure that the glabella is in a proper position in rhinoplasty practice.

Among all comparisons, nasofacial angle had the smallest effect size and nasofrontal angle had the largest. Because the width-to-length ratio depends on alar width and nasal length, it can be concluded that out of six originally measured aesthetic features, only two were different between cases and controls, and the effect sizes were moderate for these two features.

The case group and the control group were matched in demographic characteristics and did not differ in facial aesthetic proportions very much. What motivates the cases to apply for a rhinoplasty yet the controls are not interested? Hereby, the importance of psychological variables is identified. Disturbed body image, 14 lowered self-esteem, 20 depressive symptoms, 19 body dysmorphic ideation, 46 47 and other psychological variables may play a central role in the interest in aesthetic rhinoplasty. As a result, the psychological evaluation of patients desiring rhinoplasty seems required to screen potentially disturbed patients. Yet, the facial aesthetic proportions may be quite beneficial for longitudinal research designs investigating nasal shape. 41 As a result, future research may investigate the moderating roles of psychological constructs in the relationship between facial aesthetic proportions and interest in rhinoplasty.

Some limitations of the present study are worth noting. First, there are no empirically confirmed norms for facial aesthetic proportions in large populations. Comparing the proportions with locally standardized norms, instead of a control group, could have strengthened the results. Second, no self-report assessment was performed. Incorporating psychometric instruments could have enabled more advanced statistical analyses.
